# Topological edge states in high-temperature superconductiving FeSe/SrTiO_3_ films with Te substitution

**DOI:** 10.1038/s41598-019-40644-0

**Published:** 2019-03-11

**Authors:** Li Chen, Hongmei Liu, Chuan Jiang, Changmin Shi, Dongchao Wang, Guangliang Cui, Xiaolong Li, Qiandong Zhuang

**Affiliations:** 1 Institute of Condensed Matter Physics, Linyi University, Shandong, 276000 China; 2 Department of Data Acquisition, National Instruments, Shanghai, 201204 China; 3 Physics Department, Lancaster University, Lancaster, LA1 4YB UK

## Abstract

Using first principles theory, we investigated the behavior of the one-dimensional (1D) topological edge states of high temperature superconductiviing FeSe/SrTiO_3_ films with Te atoms substitution to Se atoms in the bottom (top) layer in single-layer FeSe, as a function of strain. It was discovered that the 1D topological edge states are present in single-unit-cell FeSe film on SrTiO_3_, but are absent when more than 50% Se atoms are replaced by Te atoms. Stress induced displacive phase transformation exists in FeSe/SrTiO_3_ film when Te atoms substitute Se atoms in the bottom (top) layer in single-layer FeSe under 3% strain respectively. The 1D topological edge states are present under 3% (1.8%) strain in FeSe/SrTiO_3_ films with Te substitution Se in the bottom (top) layer in single-layer FeSe, even up to 5%, respectively. This indicates that the bonding angle of Se-Fe-Se (Te) and the distance of Te (or Se) atoms to the Fe plane are correlated with the topological edge states. Our findings provide an effective interface system that provides both superconducting and topological states, opening a new route for realizing 2D topological superconductors with proximity effect.

## Introduction

Single-unit-cell (UC) FeSe films have been successfully grown on SrTiO_3_ (STO) substrate with a superconducting gap up to 20 meV^1^ and possible superconducting transition temperature above 100 K^2^. The Fermi surface of single-layer FeSe/STO have been measured by angle-resolved photoemission spectroscopy (ARPES) experiments, showing electron pockets at the M point and no hole pockets at the Γ point. This experimental observation is inconsistency with theory in band structure. Despite intense experimental^3–9^ and theoretical efforts^9,10^ have been devoted, some of the essential fascinating new-style class of 2D superconductor is still elusive. Recently, much attention has been paid to the potential being of topological phases in FeSe^11–13^. Theoretical and experimental results have presented for an antiferromagnetic (AFM) quantum spin Hall (QSH) state in FeSe^14^. Previous low-temperature scanning tunneling microscopy (STM) and scanning tunneling spectroscopy (STS) study have demonstrated that the superconducting transition temperature (Tc) increases with the substitution of Se composition by Te which reaches 14.5 K at about 40% Se substitution^15,16^, which is higher than the maximum Tc ~9 K of bulk FeSe. 1UC FeTe_1−*x*_Se_*x*_ films (0 < *x* < 0.6) on STO(001) substrates were prepared by molecular beam epitaxy (MBE) and the superconducting properties were studied by *in situ* STS study and *ex situ* transport measurement^17^.

Considering the fact of both theoretical and experimental results for an AFM QSH state in FeSe^14^, it’s necessary to test the potential existence of topological phases in high-temperature superconductiving FeSe/STO films with Te substitution to Se. The properties of FeSe films with Te substitution could be affected by the key factors including interfacial strain, structural deformation, interfacial charge transfer and so on. However, the interfacial strain is the most dominant factor amongst these. The in-plane lattice constant of FeSe exactly matches the substrate with a perfect coherent interface. While Se atoms are substituted by Te, strain will be induced to the film, so that strain is suggested to play an important role in affecting the interaction of topological band states.

Motivated by these recent discoveries, we have carried out a systematic study of the topoelectronic properties of 1UC FeSe/STO films with Te substitution as a function of concentration change of Te substituting and strain, based on first-principles calculations. More than 50% Te substitution Se atoms in both top and bottom layers in single-layer FeSe will lead to the disappear of the 1D Dirac edge state. These calculation results allow us to conclude that tensile strain induces displacive phase transformation when Te atoms substitute Se atoms in the bottom (top) layer in single-layer FeSe under 3% strain respectively. 1D topological edge states of 1UC FeSe/STO films with Te substituting Se on bottom (top) layer is presented under tensile strain 3% (1.8%), respectively, even up to 5% strain. This study provides a good foundation for experimental study that leads to potential applications in spintronics.

## Methods

**Figure 1:**
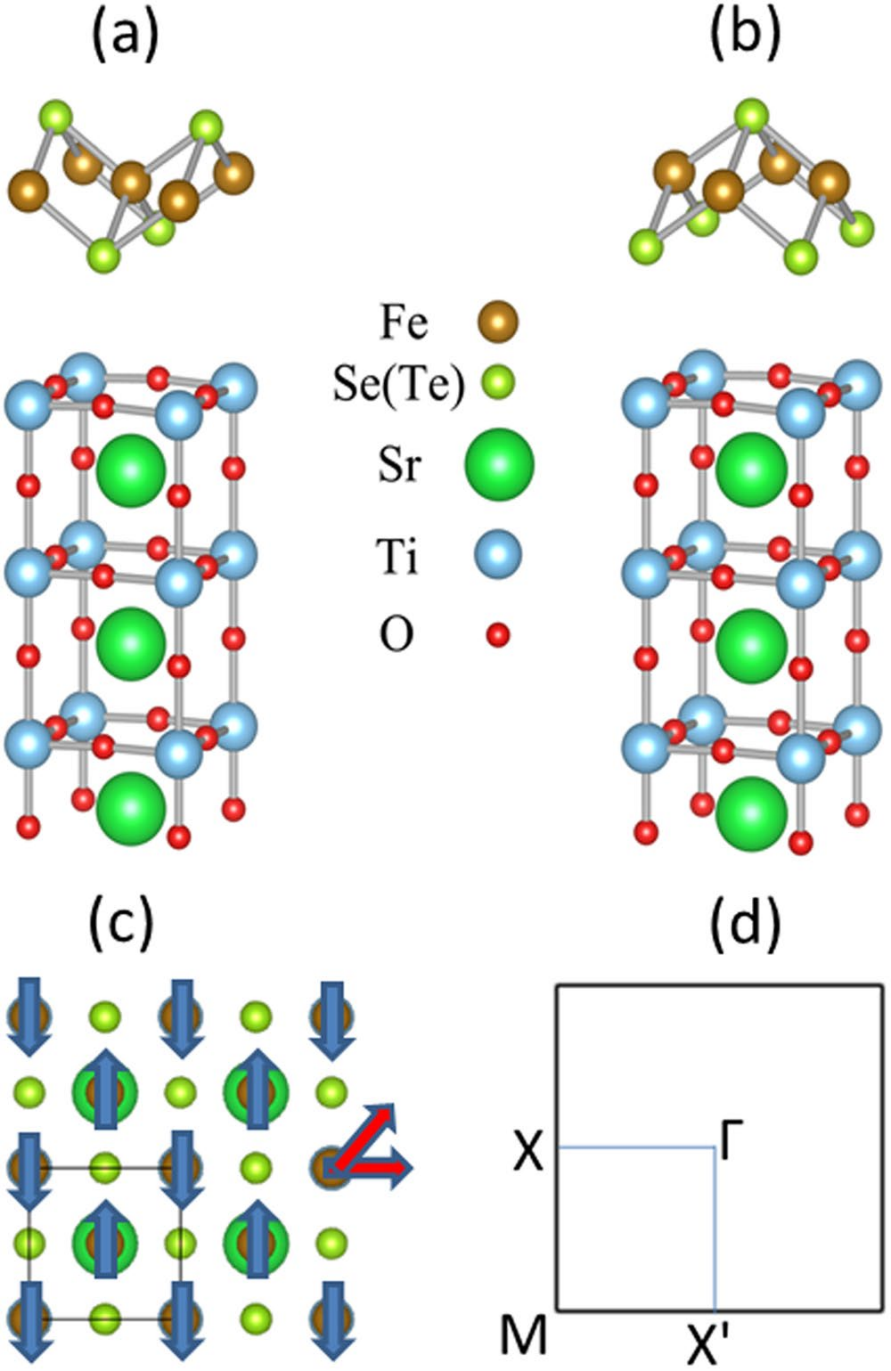
Unit cell of FeTe on SrTiO_3_ subtract with six atomic layers: (**a**) the bottom Se atom in single-layer FeSe is directly above the top O in the STO substrate, and (**b**) the bottom Se atom in single-layer FeSe is directly above the top Ti atom in the STO substrate, brown, light green, red, dark green and blue balls represent Fe, Se(Te), O, Sr and Ti atoms, respectively. (**c**) Fe atoms with chequerboard AFM spin configuration. Spin in z direction is the normal to FeSe plane denoted by blue arrows. Spin in x direction is along next-nearest-neighbor Fe-Fe bond and xy direction is along nearest-neighbor Fe-Fe bond denoted by red arrows. (**d**) Brillouin zone of unit cell of FeTe on SrTiO_3_ subtract with high symmetry point Γ, X, X′ and M.

The experimental interface configuration of FeSe/STO is complex. There are two possible atomic structures of FeSe/STO: the bottom Se atom in single-layer FeSe is directly above the top of either O or Ti atom in the STO substrate as can be seen in Fig. 1(a,b), respectively. These two interface configurations of FeSe/STO lead to equivalent result^14^. In our calculation, we assumed that the bottom Se atom in single-layer FeSe is directly above the top of O atom in the STO substrate. Its electronic band structures and topoelectronic properties were calculated with the framework of the PBE-type generalized gradient approximation using VASP package as described before^18–21^. Spin orbit coupling (SOC) was included in the self-consistent electronic structure calculations. All calculations were implemented with a plane-wave cutoff of 400 eV on a 9 × 9 × 1 Monkhorst-Pack k-point mesh, and a vacuum layer that is over 15 Å to avoid the interaction between neighboring slabs. For structural relaxation, all the atoms are allowed to relax until atomic forces are smaller than 0.01 eV/Å. The experimental lattice constant is a = 3.90 Å^22^. In order to test the influence of SrTiO_3_ antiferrodistortion on surface electronic structures, we chosen 2 × 2 × 1 supercell. Previous study indicated that the band structure of FeSe/STO with chequerboard AFM configuration coincides exactly with the ARPES spectra within the whole Brillouin zone, so that we chosen chequerboard AFM configuration in our calculations (neighbouring Fe atoms have opposite spin directions). Figure 1(c) shows the spin configurations of the chequerboard AFM state that are schematically along z, x and xy, respectively. z direction is the vertical normal to FeSe plane, x direction is along next nearest-neighbor Fe-Fe bond, and xy direction is along nearest-neighbor Fe-Fe bond. Our calculations shown that the differential energies among above three spin configurations are very small. On the other hand, previous work has proved that the in-plane spin configuration cannot leads to a sizeable gap at the M point in FeSe/STO^14^, so we set the z spin direction for Fe atoms in our calculation. We used the Wannier90 package^23^ to calculate the topoelectronic edge states of 1D zigzag nanoribbon as described previously^20,21^. The tight-binding Hamiltonian with maximally localized Wannier functions (MLWFs) was fitted to the first-principles band structures. The Coulomb repulsion is characterized by a spherically averaged Hubbard parameter U, which depends on the spatial extension of the wave functions. In our calculation the U was set to be 0.6 eV^14^. Considering that GGA is usually regarded to underestimate the binding in weakly bonded systems of inhomogeneous systems, we used van der Waals (vdw) correction in our calculations and applied the semiempirical DFT-D2 method proposed by Grimme^24^.

## Results and Discussion

We first modeled the interface structure of FeSe monolayer on TiO_2_-terminated SrTiO_3_ (001) surface in six atomic layers, where the bottom Se atom in single-layer FeSe is directly above the top O atom in the STO substrate. The vertical distance between O and Se atoms is 3.188 Å. The bond length between top (bottom) Se and Fe atoms is 2.389 (2.387) Å. The Fe-Se-Fe angle for top (bottom) Se atoms is 109.338° (109.520°). The calculated magnetic moment is 2.37 μ_B_ on each Fe atom.

**Figure 2:**
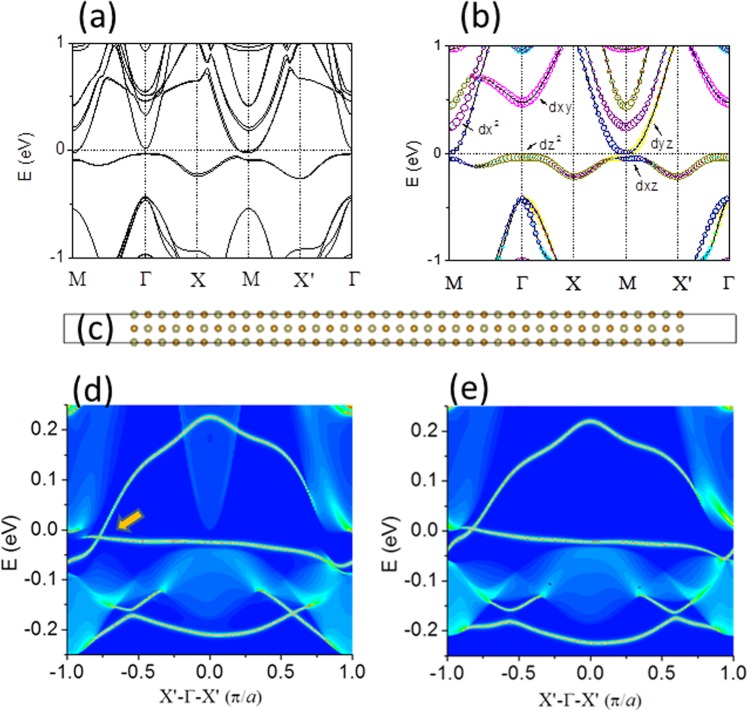
Band structures long M- Γ- X- M- X′- Γ directions (**a**) for 1UC FeSe/SrTiO_3_ film, (**b**) for free standing 1UC FeSe film. (**c**) Atomic configuration of a 1D FeSe/STO ribbon. (**d**) 1D band structure for c. (**e**) 1D band structure for free-standing 1UC FeSe film.

Figure 1(d) shows the supercell brillouin zone with high-symmetry k points including Γ, M, X and X΄. In our calculations, the monolayer lattice constants *a* and *b* of FeSe/Te on STO are same. Duo to the effect of substrate, the symmetry of this system would be lowered, the electronic structures maybe have a small difference along MX and MX΄ directions. The corresponding band structures with Hubbard-U and vdw corrections are shown in Fig. 2(a). An obvious characteristic is that no hole pocket at the Γ point and electron pocket at the M point in FeSe/STO system, which is in agreement with the ARPES data^4^ and previous calculations^14,22^. The outspread flat bands below the Fermi level come from the d_z2_, d_xz_ and d_yz_ orbitals of the Fe atoms, and the p_z_ orbital of the Se atoms. We demonstrate the band of free standing 1UC FeSe monolayer in Fig. 2(b) to compare the energy bands with substrate effects, thus further examining the influence of the substrate on the band. The STO substrate enhances the feature of electron pockets at the M point, showing that the hybridization between FeSe and STO substrate is negligible and the parabolic bands overlapping with the FeSe bands are most from the STO substrate.

1D ferromagnetic (FM) edge states of a FeSe/STO ribbon indicated in Fig. 2(c) were studied using the Wannier functions^25–27^. Spin symmetry breaking along the FM edge leads to two asymmetric edge states for both the left and right edges. The edge states with bright lines are presented around Fermi level and Dirac point with the valence and conduction bands connecting is appeared in Fig. 2(d). Most importantly, the pair of gapless Dirac edge states inside the SOC gap demonstrates AFM QSH state. Figure 2(e) shows the edge states of free standing FeSe, exhibiting 1D Dirac edge state inside the “bulk” band gap. This verifies that the 1D Dirac edge states of 1UC FeSe/STO originate in the 1UC FeSe. Compared with Fig. 2(d), the interaction between SrTiO_3_ substrate and FeSe monolayer induces charge transfer and an interfacial electric field, which slightly modify the relative shape in “bulk” band structures while keep Dirac edge state.

1UC FeTe_1−*x*_Se_*x*_ films (0 < *x* < 0.6) on STO (001) substrates were reported with superconducting properties examined by combined *in-situ* STS study and *ex-situ* transport measurements^17^. In experiments, atom doping is an out of order distribution. The simulation modeling of such disorder is very complicated and time consuming. For simplicity, we firstly assumed that the Te replaces either the bottom or top Se atoms in 1UC FeSe. In this substitution we could distinguish which one between Se and Te atoms is benefit to induce 1D Dirac edge state in this system. Firstly, we consider that Te atoms completely substitute bottom Se atom in single-layer FeSe (The system is denoted as FeSe_Te_/STO, see Supplementary Fig. 1(a)). The vertical distance between O and Te atoms is 3.328 Å. This confirms that the Te atom substituting Se atom lowers the interaction between FeSe_Te_ and STO substrate. The bond length between top (bottom) Se (Te) and the Fe atoms is 2.368 (2.542) Å. The Fe-Se (Te)-Fe angle for top Se and (bottom Te) atoms is 107.66° (97.92°). The calculated magnetic moment is 2.23 μ_B_ on each Fe atom. Te atom substituting changes bond length and bond angle with Fe atom, and decreases interaction in FeSe_Te_/STO interfacial system slightly.

**Figure 3:**
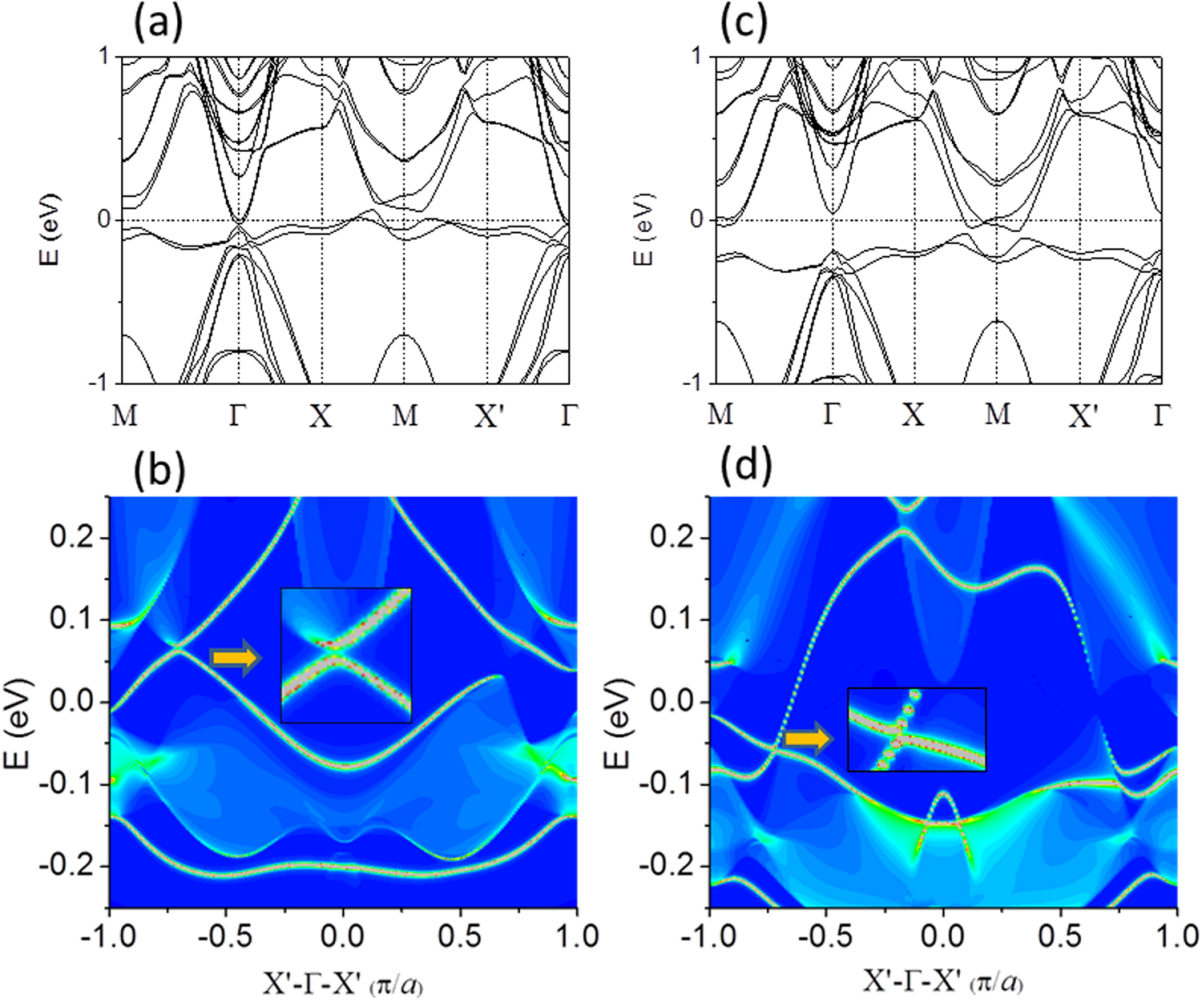
Band structures long M- Γ- X- M- X′- Γ directions (**a**) for 1UC FeSe_Te_/SrTiO_3_ film with Te atom substituting bottom Se atom in single-layer FeSe. (**b**) 1D band structure for a. (**c**) For 1UC FeSe^Te^/SrTiO_3_ film with Te atom substituting top Se atom in single-layer FeSe. (**d**) 1D band structure for c.

The bands for FeSe_Te_/STO film are shown in Fig. 3(a). We could see that the band at Γ point shows electron pocket, which is the typical dz^2^ feature, whereas the bands along X- M line still show hole pockets, which are the dxz and dyz features. The Te substitution enhances the feature of electron pockets at Γ point slightly. In addition, this Te substitution induces the spatial inversion symmetry breaking. This is similar to the external electric field induced Rashba band splitting in the surface state of system. The 1D edge states of a FeSe_Te_/STO ribbon with FM edge shown in Fig. 3(b) were calculated using the Wannier functions. The valence and conduction bands is not connecting with a pair of gapless Dirac edge states in interior illustration to guide eyes, which could be seen in Fig. 3(b).

Subsequently, we considered Te atom substituting Se atom on the top layer of FeSe (denoted as FeSe^Te^/STO, as indicated in Supplementary Fig. 1(b)). The vertical distance between O and Se atoms is 3.217 Å, which is the less than that between O and Te atoms in FeSe_Te_/STO by 0.111 Å. The bond length between top (bottom) Te (Se) atoms and the Fe atom is 2.53 (2.36) Å. The Fe-Te(Se)-Fe angle for top Te and (bottom Se) atoms is 97.92° (107.66°). The calculated magnetic moment is 2.24 μ_B_ on each Fe atom which is almost same as 2.23 μ_B_ in FeSe_Te_/STO interface system.

Comparing the bands of FeSe_Te_/STO interfacial system in Fig. 3(a,c), we can realize an obvious characteristic that electron pockets at the M point and no hole pocket at the Γ point seen from Fig. 3(c) for FeSe^Te^/STO interfacial system, which is similar to FeSe/STO interfacial system (Fig. 1(c)). Bands at Γ point have lower energy with increased d_z2_ orbital, except Rashba band splitting in the surface state of system. The 1D edge states FeSe^Te^/STO ribbon with FM edge were also calculated using the Wannier functions. The original Dirac point opens a tiny gap appeared in Fig. 3(d) for FeSe^Te^/STO ribbon. The interior enlarged view could confirm that. Supplementary Fig. 2 shows the edge states of free standing FeSe with Te atom substituting Se atom in top layer, exhibiting 1D Dirac edge state inside the “bulk” band gap. This verifies that the 1D Dirac edge states are originated in the 1UC FeSe with Te substituting and the interaction between SrTiO_3_ substrate and FeSe^Te^ monolayer induces original Dirac point opening a tiny gap.

**Figure 4:**
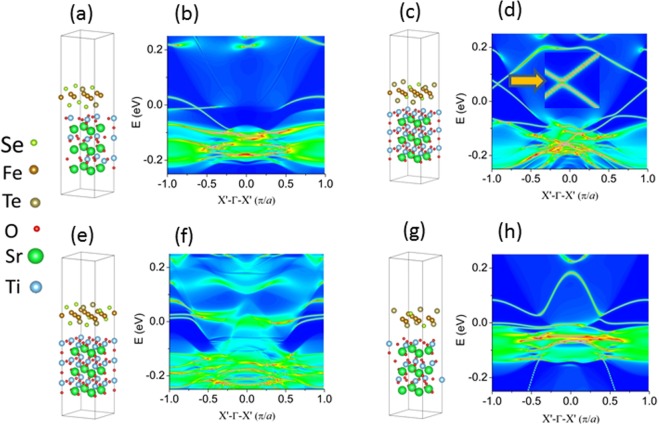
(**a**) 2 × 2 × 1 supercell of FeSe_0.75_Te_0.25_/SrTiO_3_ film and (**b**) 1D band structure for a; (**c**) 2 × 2 × 1 supercell of FeSe_0.50_Te_0.50_/SrTiO_3_ film and (**d**) 1D band structure for c; (**e**) 2 × 2 × 1 supercell of FeSe_0.50_Te_0.50_/SrTiO_3_ film and (**f**) 1D band structure for e; (**g**) 2 × 2 × 1 supercell of FeSe_0.25_Te_0.75_/SrTiO_3_ film and (**h**) 1D band structure for g.

Next we focused on the effect of Te substitution concentration (variation of *x*) on the band details. In our calculation, we assumed that Te atoms substitute Se atoms (on both top and bottom layers) with 0.25%, 50%, 50% and 75% concentration in single-layer FeSe of 2 × 2 × 1 supercell as shown in Fig. 4(a,c,e,g), respectively. Among these four atomic configurations, Fig. 4(c,e) are with same substitution concentration but with different atomic configurations. Figure 4(b,d,f,h) present the 1D band structure for Fig. 4(a,c,e,g), respectively. Figure 4(b) shows the edge states of FeSe_0.75_Te_0.25_/STO, exhibiting 1D Dirac edge state inside the “bulk” band gap, while others do not show the Dirac edge states. Figure 4(d) indicates the original Dirac point opens a tiny gap, which is in interior illustration to guide eyes. Our calculations confirm that Te atomic substitution more than 50% will lead to the disappearing of the original Dirac point due to the local structure change induced spin-up and spin-down channels coupling from each other.

**Figure 5:**
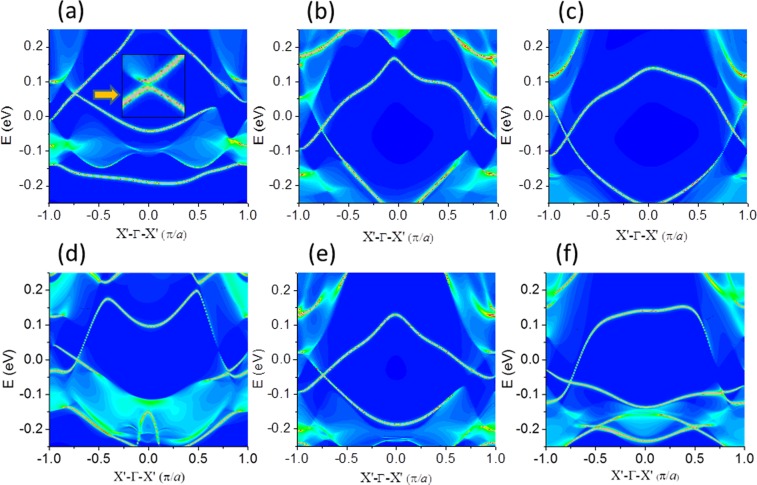
1D band structure for 1UC FeSe_Te_/STO film (**a**) under 1.8% strain with *a* = 3.972 Å, (**b**) under 3% strain with *a* = 4.018 Å, (**c**) under 5% strain with *a* = 4.096 Å. 1D band structure for 1UC FeSe^Te^/STO film (**d**) under 1.8% strain with *a* = 3.972 Å, (**e**) under 3% strain with *a* = 4.018 Å, (**f**) under 5% strain with *a* = 4.096 Å.

Experimental and theoretical analysis reveal there are two key effects dominant the properties of the system. The first is the Rashba effect introduced by internal electrical field with interfacial charge transfer when the films are grown on the substrate^21,28,29^. The second is the strain effect on the thin film. The thin film can form a perfect coherent interface with the substrate^21,28,29^ so that strain plays an important role in affecting the topological band states in addition to internal electrical field effect^29^. While generally they may be considered as negative factors, we may also work them to our advantage. In fact, strain and interfacial engineering have already become a common strategy to modulate the properties of thin films^30^. It is anticipated that introducing tensile biaxial strain will tune the helical Dirac points existing in the gap of bulk bands. As shown in Fig. 5(a), the two bands below the Fermi level between X′- Γ are nearly cross with the increasing tensile biaxial strain in FeSe_Te_/STO ribbon while the strain is under 1.8% (lattice constant *a* = 3.972 Å). When the strain increases to 3% (with lattice constant *a* = 4.018 Å), e.g. the bond length between Te (Se) atoms and the Fe atom is 2.57 (2.40) Å and the Fe-Te(Se)-Fe angle is 102.90° (113.41°). The interfacial distance decreases to 2.943 Å with an increased interface energy of 0.05 eV. The decreased interfacial distance and the increased interface energy enhance the interfacial coupling. In particular, stress-induced displacive phase transformation could be found while the thin film is under a strain of 3%, where the bottom Te atom in single-layer FeSe is directly above the top Ti atom in the STO substrate (see Fig. 3(a) in Supplementary). Band structures long M- Γ- X- M- X′- Γ directions under 3% strain show that electron pockets along X- M line and no hole pocket at the Γ point (see Fig. 4(a) in Supplementary). 1D Dirac edge states of FeSe_Te_/STO ribbon occurs under this strain as presented in Fig. 5(b). More importantly, under a strain of 5% (lattice constant *a* = 4.096 Å), two Dirac edge points could formed in 1D band structure of 1UC FeSe_Te_/STO film as shown in Fig. 5(c). The position of VBM and CBM connect between X′- Γ, forming a spin-polarized Dirac point inside the “bulk” band gap. Therefore, the topological Dirac states and their peculiar properties could be accessed by quantum control. We would emphasize that the increase of interfacial coupling induces Dirac states. Te substituting Se breaks the symmetry of system. The position where the band gap closes in the Brillouin zone depends on the symmetry of the system. In an inversion symmetric system, the gap closes at time-reversal invariant momenta, such as the Γ point, while in an inversion asymmetric system the gap vanishes at other points instead of at time-reversal invariant momenta. Similarly, stress-induced displacive phase transformation of FeSe^Te^/STO film is also found under a strain of 3% (see from Fig. 3(b) in Supplementary), where the bond length between Te (Se) atoms and the Fe atom is 2.58 (2.40) Å and the Fe-Te(Se)-Fe angle is 102.46° (113.98°). The interfacial distance decreases to 2.866 Å with an increased interface energy of 0.24 eV. Band structures (Supplementary Fig. 4(b)) long M- Γ- X- M- X′- Γ directions under 3% strain show that electron pockets along X- M line and hole pocket at the M point. For the strained FeSe^Te^/STO ribbon, their corresponding band structures are shown in Fig. 5(d–f) under a strain of 1.8%, 3% and 5%, respectively. The 1D edge states appear as the strain 1.8% case, except for the relative shape change. But there is no Dirac edge state within the band gap (see Fig. 5(a)) under same strain for FeSe_Te_/STO ribbon. Therefore, the FeSe^Te^/STO ribbon remains 1D Dirac edge states under less strain conditions, even up to 5% strain. The atomic structure changing under the strain results in the different shape of the edge states as shown in Fig. 5.

**Figure 6:**
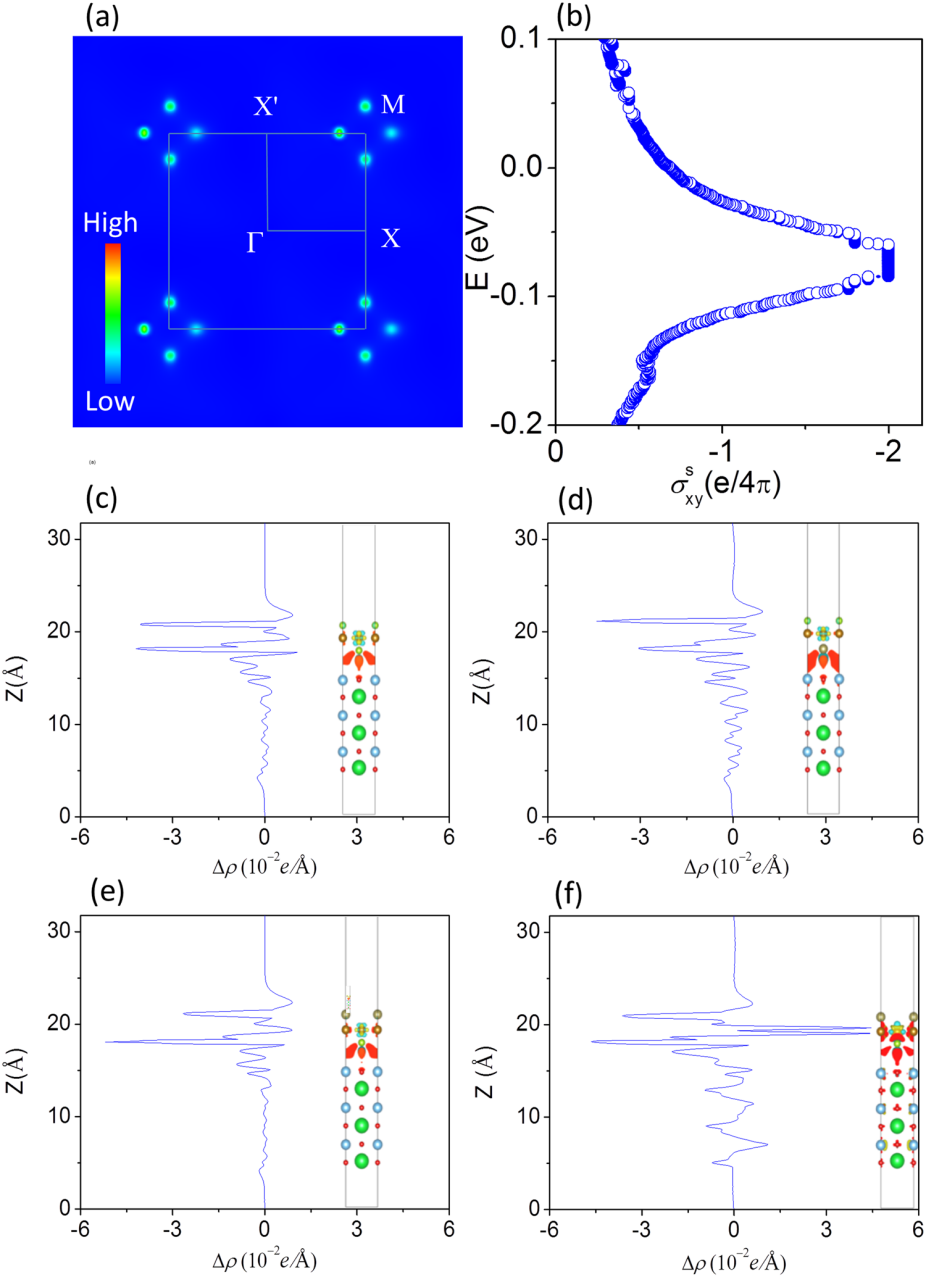
(**a**) Spin Berry curvature within the SOC gap in reciprocal-space. (**b**) Spin Hall conductance as a function of the Fermi level within SOC gap. Differential charge density (**c**) corresponding 1UC FeSe/SrTiO_3_ film (**d**) corresponding 1UC FeSe_Te_/SrTiO_3_ film, (**e**) corresponding 1UC FeSe^Te^/SrTiO_3_ film. (**f**) Corresponding 1UC FeSe^Te^/SrTiO_3_ film with 1.8% strain. The charge density isosurface is set to be 0.0005 e/Å^3^.

The time-reversal symmetry is preserved in the conventional QSH state while it is broken in the AFM system. However, AFM QSH state is presented if spin-up and spin-down channels are decoupled from each other. 3D AFM topological insulators^31,32^ has been studied under the combined symmetry of time-reversal and primitive-lattice-translation. On the basis of the Bernevig_Hughes_Zhang model with an AFM stagger potential, the edge states in the AFM QSH state are still robust against non-magnetic disorder^33^ though the non-magnetic disorder can break this combined symmetry. Berry Curvature and Chern number are the key factors to determine whether the gap induced by SOC is topological nontrivial. The Berry curvature Ω (k) is calculated by the equation^34,35^

1 $${\rm{\Omega }}(\overrightarrow{k})=\sum _{n}\,{f}_{n}{{\rm{\Omega }}}_{n}(\overrightarrow{k})$$

2 $${{\rm{\Omega }}}_{n}(\overrightarrow{k})=-\sum _{n^{\prime} \ne n}\,2Im\frac{\langle {\psi }_{nk}|{\upsilon }_{x}|{\psi }_{n\text{'}k}\rangle \langle {\psi }_{n^{\prime} k}|{\upsilon }_{y}|{\psi }_{nk}\rangle }{({\varepsilon }_{n^{\prime} k}-{\varepsilon }_{nk})}$$

3 $${{\rm{\Omega }}}^{s}(\overrightarrow{k})=\sum _{n}\,{f}_{n}{{\rm{\Omega }}}_{\,n}^{s}(\overrightarrow{k})$$

4 $${{\rm{\Omega }}}_{\,n}^{s}(\overrightarrow{k})=\,-\sum _{{n}^{\text{'}}\ne n}\,2Im\frac{\langle {\psi }_{nk}|{j}_{x}|{\psi }_{n\text{'}k}\rangle \langle {\psi }_{n\text{'}k}|{\upsilon }_{y}|{\psi }_{nk}\rangle }{({\varepsilon }_{n^{\prime} k}-{\varepsilon }_{nk})}$$

where *f*_*n*_ is the Fermi distribution, *ψ*_*nk*_ is the eigenstate of eigenvalue ${\varepsilon }_{n^{\prime} k}$ of band n, ${\nu }_{{\boldsymbol{x}}}/{\nu }_{{\boldsymbol{y}}}$ is the velocity operator. We use the WANNIER90 package to calculate the Berry curvature for the whole valence bands along the high-symmetry directions. The calculated spin Berry curvature for the Fermi level within the SOC gap is non-zero around four M points in reciprocal-space as can be seen from Fig. 6(a) for 1UC FeSe_Te_/STO film under 3% strain with a = 4.018 Å. The Chern number (C) is expressed as

3 $${\rm{C}}=\frac{1}{2\pi }{\int }_{{\rm{BZ}}}{d}^{2}\overrightarrow{k}{\rm{\Omega }}(\overrightarrow{k})$$

4 $${\rm{C}}={C}_{\uparrow }+{C}_{\downarrow }$$

5 $${C}^{s}=\frac{1}{2}({C}_{\uparrow }-{C}_{\downarrow })$$

Where *C*^*s*^ is spin Chern number and it is expressed as

6 $${C}^{s}=\frac{1}{2\pi }{\int }_{{\rm{BZ}}}{d}^{2}\overrightarrow{k}{{\rm{\Omega }}}^{s}(\overrightarrow{k})$$

By integrating the Berry curvatures over the first Brillouin zone (BZ), we can gain the spin Chern number with −1 and then the spin Hall conductance can be obtained from the spin Chern number as

7 $${\sigma }_{xy}^{SH}=\frac{e}{4\pi }({C}_{\uparrow }-{C}_{\downarrow })$$

Correspondingly, an AFM QSH state is confirmed with quantized value of spin Hall conductance within the energy range of the SOC gap in Fig. 6(b). In a FeSe/Te monolayer grown on STO substrate, the spatial inversion symmetry is indeed already broken, but the topological edge state still exists because of the finite coupling between the monolayer and the substrate where spin-up and spin-down channels are decoupled from each other. When Te replaces the bottom or top Se atoms in 1UC FeSe this disorder changes the electric potential and decreases the spin degeneracy of the AFM state, hence increases the gap of spin-split band, leading to the open of Driac edge state. Tensile strain will destroy coupling between spin-up and spin-down channels. When spin-up and spin-down channels are decoupled from each other again under large tensile strain, the Driac edge states present again.

It was found that the QSH state characterized by nonzero spin Chern numbers ±1 persists when the time reversal symmetry is broken with exchange field^36^. The quantum spin Hall phase could exist until the exchange field strength reaches a critical value. When the bulk band gap closes and then opens, the system enters the quantum anomalous Hall phase^37^ characterized by 1 (or −1). The non-trivial spin Chern number requires the existence of an edge state at the sample boundary without considering any symmetry^36^. Spin Chen Number is an effective tool to characterize the different topological phases of quantum spin Hall system without time inversion symmetry.

Although the superconducting properties of 1UC FeSe/STO films originate in the 1UC FeSe film without Te or with Te substituting, the STO substrate provides possibly a triggering mechanism that induces the superconducting states in the systems. When the FeSe film is attached on SrTiO_3_ substrate, local tension exists at the interface of heterojunction materials, which leads to charge transfer. This leads to a Rashba effect that is resulted from the internal electrical field which is introduced by interfacial charge transfer when they are grew on the STO substrate. The differential charge density at the interface is defined as *ρ* = *ρ*_FeSe/STO_ − *ρ*_FeSe_ − *ρ*_STO_, where, *ρ*_FeSe/STO_, *ρ*_FeSe_, and *ρ*_STO_ are the charge density of FeSe/STO respectively, and FeSe and STO are at the same surface lattice constant. The differential charge density at the interface of FeSe/STO film is presented in Fig. 6(c). Our Bader analysis identifies a charge transfer of 10^−3^ e per cell from STO substrate to FeSe film. In practice, the actual charge transfer is larger because defects in substrate results in additional charge transfer. The differential charge density at the interface of two mentioned systems FeSe_Te_/STO and FeSe^Te^/STO are presented in Fig. 6(d–f). It confirms that Te atoms have less ability to acquire electrons in comparison with Se atoms, resulting in a decreased electron-phonon coupling. This might be one of the reasons that 1UC FeSe/STO film exhibits a superconducting state, while 1UC FeTe/STO doesn’t. Figure 6(f) also shows that FeSe^Te^ monolayer has a different charge density due to more electrons transfer from Fe atoms to Se and Te under 1.8% strain. This is confirmed in our Bader analysis showing a charge transfer of 0.03 e from Fe atom to Se and Te atoms. This charge transfer generates an internal electric field at the interface region. The electric field leads to bands along M-X and M-X′ directions with an obvious band splitting around M point. The amplitude of this splitting is enhanced in FeSe^Te^/STO and FeSe_Te_ /STO films than that in FeSe/STO. In order to further verify the charge transfer and dipole effect of SrTiO_3_ substrate, we also calculated electronic structure for a free-standing monolayer FeSe^Te^ (see Fig. 5 in Supplementary). Our calculation clearly reveals that the splitting of energy bands decreases along M-X and M - X′ directions without the presence of electric field.

The strain-induced different Rashba states, that originate from their different response to strain, can be understood from deformation potential theory^38,39^. The surface state is presented by defining the surface deformation potential^40^

8 $${\rm{\Xi }}=({\rm{\partial }}{E}_{{\rm{F}}})/{\rm{\partial }}\varepsilon $$

where *E*_F_ and *ε* represent the Fermi energy and the applied strain, respectively. As Fermi energies of different strained systems are different, the deformation potential of the band in FeSe^Te^/STO system is smaller than that in FeSe_Te_/STO system. This indicates that the 1D band of the FeSe^Te^/STO system is more sensitive to strain than that in FeSe_Te_/STO system. Therefore, FeSe^Te^/STO system is more superior and more practical than FeSe_Te_/STO system to tune the quantum phase with a spin-polarized Dirac edge states inside the band gap under tensile strain. This indicates that the bonding angle of Se-Fe-Se (Te) and the distance of Te or Se atoms away from the Fe plane are correlated with the topological edge states.

In conclusion, we have studied the electronic properties of high-temperature superconductiving FeSe/SrTiO_3_ films without and with Te substitution by first-principles theory. Our calculations show that, although the bands are changed by interface electronic field, the 1D topological edge states appear within the gap without Te substitution. Interfacial Se atom layer is beneficial to form the1D topological edge states. Te substitution to Se atoms larger than 50% in both top and bottom layers in single-layer FeSe will lead to absence of original Dirac point. Stress-induced displacive phase transformation occurs in FeSe/SrTiO_3_ films with Te substitution under 3% strain. For the case of Te submitting the Se atom on the bottom layer of FeSe, the films on SrTiO_3_ exhibit no 1D topological edge states with a strain less than 3%. For the case of Te submitting the Se atom on the top layer of FeSe, the films on SrTiO_3_ exhibit 1D topological edge states under a strain of 1.8–5%. Since the 1D topological edge states of FeSe^Te^/SrTiO_3_ film are more sensitive to strain than that of FeSe_Te_ /SrTiO_3_ film, we propose that Se atom layer should be used as a more practical interfacial atom layer on STO than Te atom layer. We also found that the bonding angle of Se-Fe-Se (Te) and the distance of Te or Se atoms away from the Fe plane are correlated with the topological edge states. These results provide an effective interface system that supports both superconducting and topological states, shedding light on realizing 2D topological superconductors on oxide substrate.

## Supplementary information

Supplementary Dataset 1
