# Supplementary material for: Topological edge states in high-temperature superconductiving FeSe/SrTiO3 films with Te substitution
Source: Sci Rep. 2019 Mar 11;9:4154. doi: 10.1038/s41598-019-40644-0 (PMC6411874; doi:10.1038/s41598-019-40644-0)
Supplement: Supplementary file 1 — Supplementary Dataset 1 [file 41598_2019_40644_MOESM1_ESM.docx]

**Supplementary Figures**

**Topological edge states in high-temperature superconductiving FeSe/SrTiO_3_ films with Te substitution**

Li Chen^1^,*, Hongmei Liu^1^, Chuan Jiang^2^, Changmin Shi^1^, Dongchao Wang^1^, Guangliang Cui^1^, Xiaolong Li^1^ and Qiandong. Zhuang^3^

^1^Institute of Condensed Matter Physics, Linyi University, Shandong 276000, China

^2^Department of Data Acquisition, National Instruments, Shanghai 201204, China

^3^Physics Department, Lancaster University, Lancaster LA1 4YB, UK


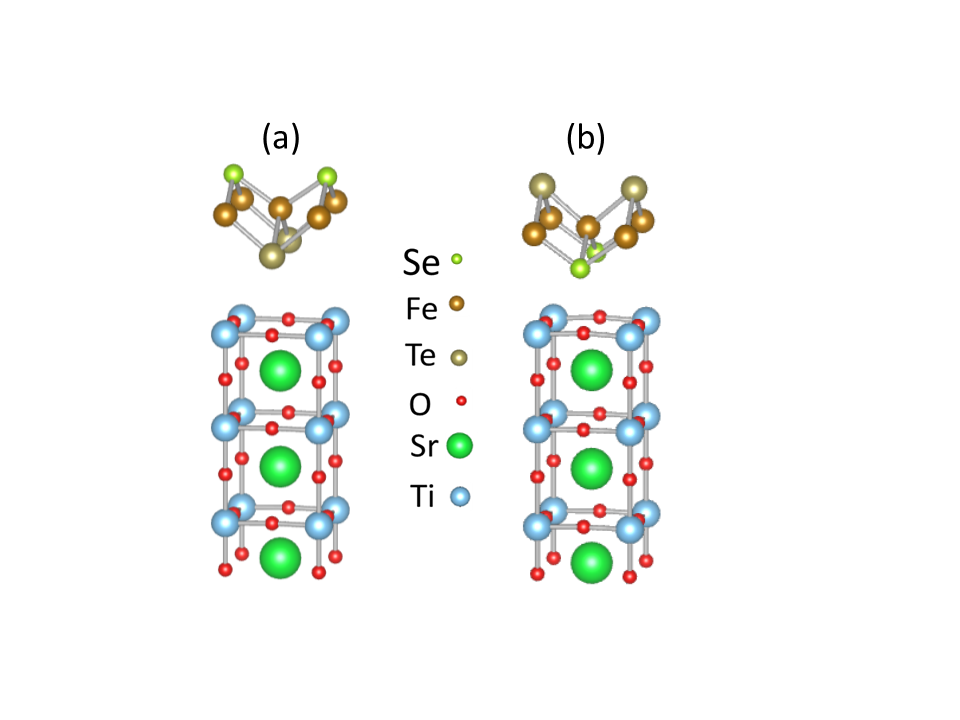


Supplementary Fig.1 (a) the bottom Te atom in single-layer FeSe is directly above the top O atom in the STO substrate. (b) the bottom Se atom in single-layer FeSe is directly above the top O atom in the STO substrate.


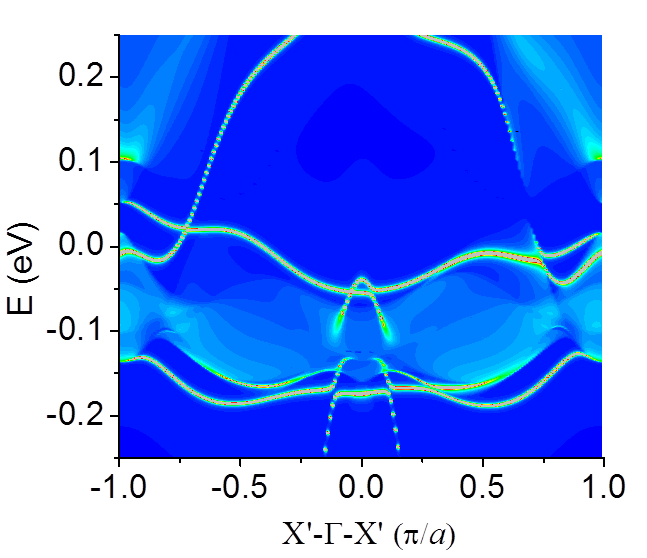


Supplementary Fig.2 The 1D Dirac edge state of free standing FeSe with Te atom substituting Se atom in top layer.


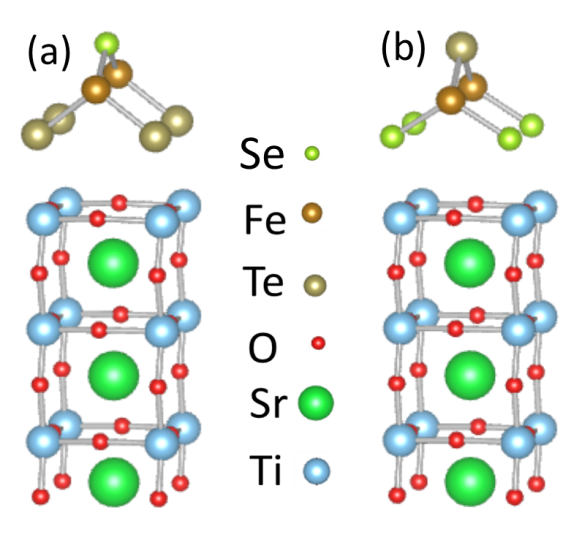


Supplementary Fig.3 Stress-induced displacive phase transformation under strain 3%. (a) The bottom Te atom in single-layer FeSe is directly above the top Ti atom in the STO substrate. (b) The bottom Se atom in single-layer FeSe is directly above the top Ti atom in the STO substrate.


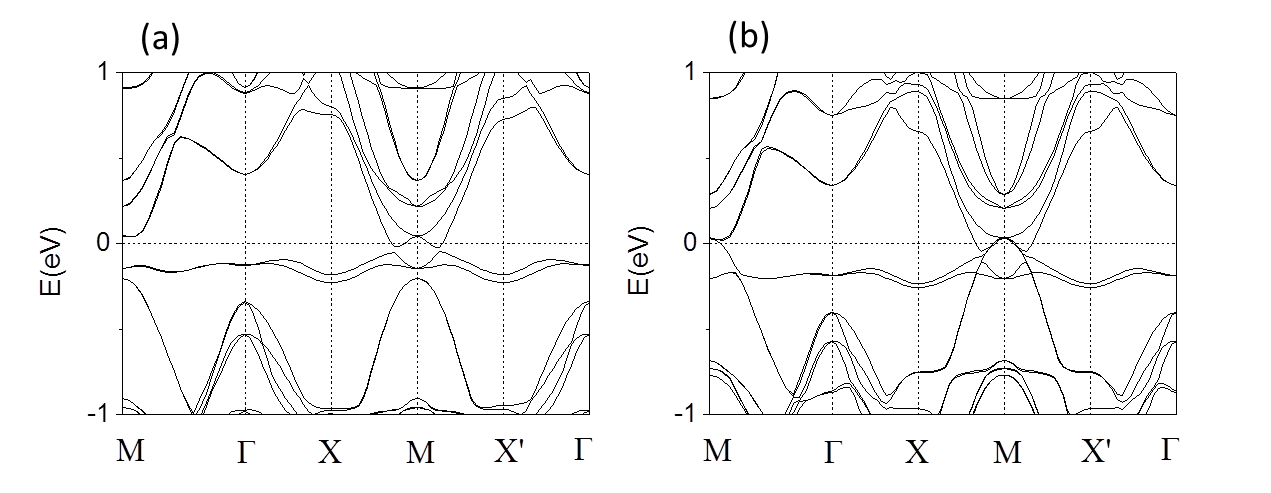


Supplementary Fig. 4 Band structures long M- Γ- X- M- X'- Γ directions under 3% strain

for 1UC FeSe_Te_/SrTiO_3_ film with Te atom substitution bottom Se atom in single-layer FeSe. (b) for 1UC FeSe^Te^/SrTiO_3_ film with Te atom substitution top Se atom in single-layer FeSe.


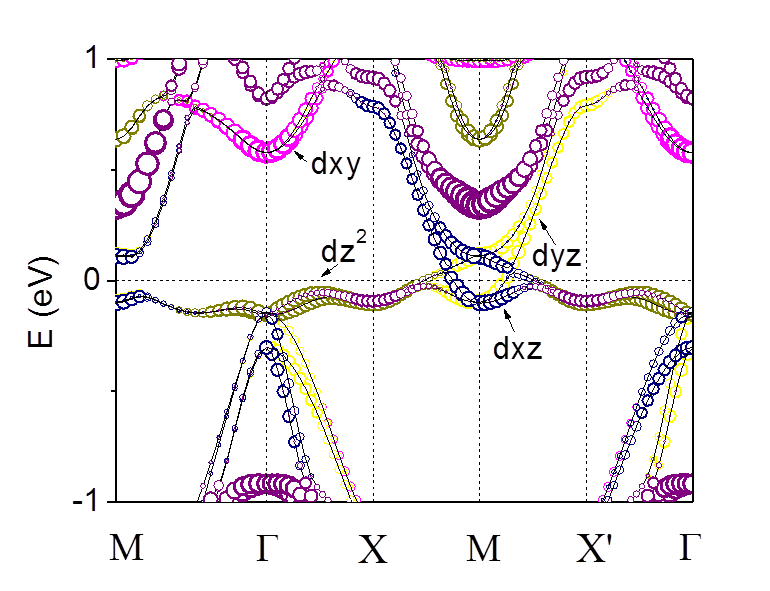


Supplementary Fig. 5 band structure for free-standing 1UC FeSe film with Te atom substitution Se atom in top layer.
